# Optic Nerve Ultrasound for Monitoring Deteriorating Intracranial Hemorrhage in a Patient on Extracorporeal Membrane Oxygenation: A Case Report

**DOI:** 10.7759/cureus.42719

**Published:** 2023-07-31

**Authors:** Christian M Renwick, Jonathan Curley

**Affiliations:** 1 Anesthesiology and Critical Care, University of Virginia, Charlottesville, USA

**Keywords:** critical care, intracranial hemorrhage, ecmo, ultrasound, optic nerve sheath

## Abstract

We present a 52-year-old male patient with cardiogenic shock who was placed on veno-arterial extracorporeal membrane oxygenation (ECMO) as a bridge to an orthotopic heart transplant. While on ECMO, the patient developed an acute intracranial bleed confirmed on computerized tomography (CT). However, his clinical status deteriorated and he was unstable for transport to evaluate for worsening hemorrhage. Instead, optic nerve sheath (ONS) ultrasonography was utilized to confirm increased intracranial pressure, which guided the goals of care until he stabilized enough to transport for advanced imaging. Repeat CT confirmed the worsening of his cerebellar bleed with obstructing hydrocephalus and brainstem compression. This case demonstrates how ONS ultrasound can be utilized in a cardiothoracic intensive care unit to evaluate sedated patients for new or worsening intracranial hemorrhage. In ECMO patients, who are often unstable with the risks of transportation for CT outweighing potential benefits, ONS ultrasonography can provide the care team with meaningful data on a patient’s neurologic status.

## Introduction

Extracorporeal membrane oxygenation (ECMO) is a vital tool for severe respiratory and circulatory failure. Unfortunately, veno-arterial (VA) and veno-veno (VV) ECMO are not without significant risk. Patients on ECMO are often coagulopathic. Combined with high-dose anticoagulation -most often unfractionated heparin - these patients have an increased intracranial hemorrhage (ICH) risk per the Extracorporeal Life Support Organization (ELSO) Registry. In the 3% of ECMO patients that develop an ICH, the mortality risk increases from 56% to 86% and 36% to 73% for VA and VV ECMO, respectively [1-2]. Comparatively, the incidence of ICH amongst the general population is roughly 0.019%, with in-hospital mortality of 32.4% [3].

As with any significant bleed, ICH is a devastating complication, associated with substantial mortality and disability. Diagnosing ICH early allows for careful decision-making of whether to pursue treatment or withhold potentially scarce treatment resources. However, effective screening for ICH is limited in ECMO patients by sedation and the inability to perform a neuro exam. Despite a non-contract computerized tomography (CT) scan of the head being the gold standard diagnostic tool for ICH, transporting ECMO patients for imaging is not without risk. Over 30% of transports are affected by adverse events, such as significant bleeds, unstable hemodynamics, or equipment malfunctions (7%, 12%, and 15% of events, respectively) [4].

Another option would be optic nerve sheath (ONS) ultrasonography, which has been validated for use in detecting elevated intracranial pressure (ICP). The ONS is particularly sensitive to increases in ICP, with an increased ONS diameter having a pooled sensitivity of 0.9 and specificity of 0.85 for elevated ICP [5]. This technique has the potential in ECMO patients to reduce the risk of travel-related events and improve treatment decision-making. As such, we present the following case describing the authors’ experience with ONS ultrasonography for guiding the management of an intracranial hemorrhage. No patient-identifying information was provided and all imaging is depicted with no patient identifiers. Therefore, this case report was exempt from informed consent.

## Case presentation

**Figure 1:**
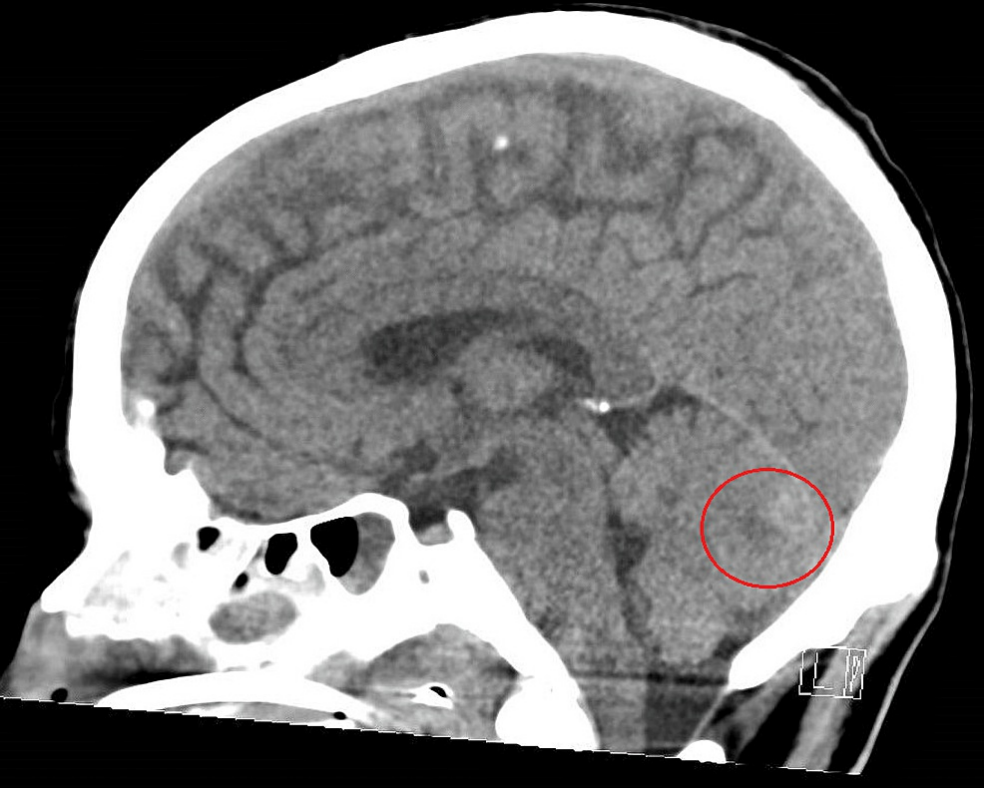
Initial CT Head Initial CT imaging obtained while the patient demonstrated agitation without gross neurologic deficit. Shown is a mid-sagittal slice. Imaging demonstrates a left cerebellar parenchymal hemorrhage (red circle, measured at 3.5 cm x 1.5 cm). Fourth ventricle remains patent. No hydrocephalus.

A 52-year-old male patient with a pertinent past medical history of myocarditis secondary to acute coronavirus disease 2019 (COVID-19) infection and heart failure with a reduced ejection fraction of 20-25% presented in cardiogenic shock. His shock state was suspected as being secondary to his non-ischemic cardiomyopathy and the patient was placed on VA ECMO via femoral artery/femoral vein approach while undergoing evaluation for an orthotopic heart transplant. The patient’s course was complicated by arrhythmias, acute renal failure requiring continuous renal replacement therapy, intracardiac thrombus formation, and bloody nasogastric output from a presumed gastrointestinal bleed. To further assess potential gastrointestinal bleeding, the patient was sent for a CT Abdomen. Up to this, point he had consistently demonstrated a normal neuro exam when sedation was held; however, a CT Head was also ordered given our intermittent inability to discontinue sedation without soliciting agitation. Sedation was maintained with propofol to limit agitation. The initial CT Head demonstrated an acute intraparenchymal hemorrhage in the left cerebellar hemisphere, measuring 3.5 cm x 1.5 cm, with partial effacement of the fourth ventricle (Figure 1). At this time, there was no hydrocephalus, intraventricular hemorrhage, or herniation, and no neurologic deficits were identified when sedation was held. Given that the patient had a known intracardiac thrombus and ongoing VA ECMO support, the decision was made to not hold his heparin anticoagulation. Neurosurgery was consulted but did not recommend surgical intervention. Hemodynamic goals remained unchanged, with a mean-arterial pressure goal of 65 - 80 mmHg.

**Figure 2:**
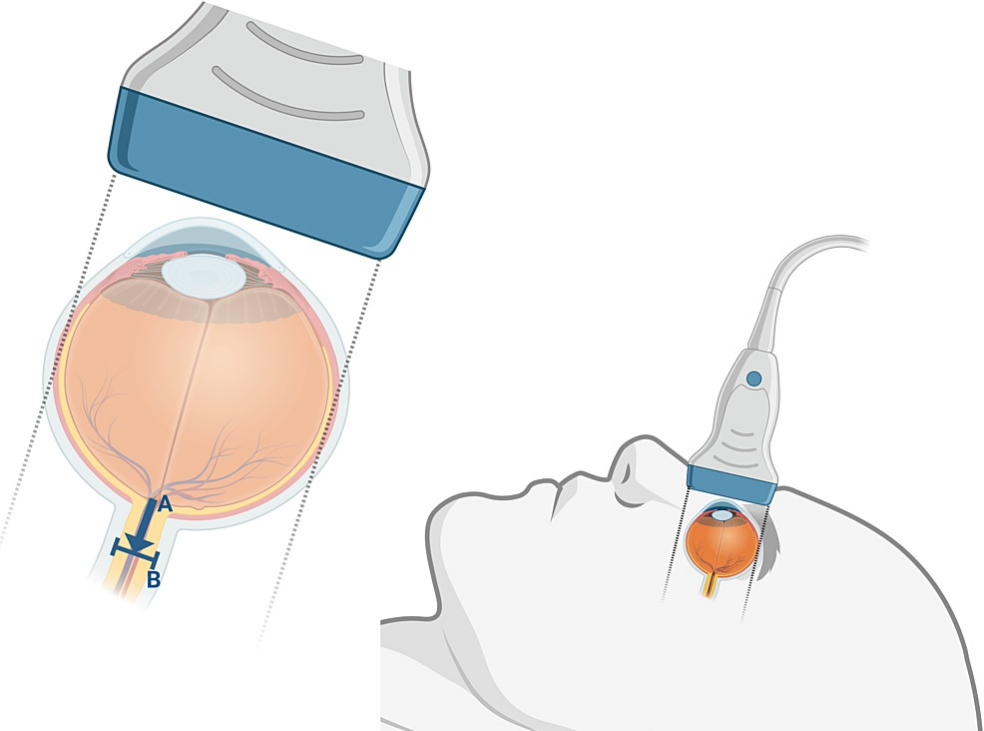
Illustration depicting the technique for measuring the optic nerve This illustration depicts the technique described for obtaining optic nerve sheath measurements. A linear probe is applied over the eye, with a Tegaderm (or another clear bandage) as a barrier between the eye and ultrasound gel. Note that the ultrasound probe should be oriented axially to the patient for the most accurate measurements. With the gain and distance optimized and the focus placed just posterior to the eye, optic nerve sheath measurements can be obtained in a mid-axial view. Distance A represents 3mm posterior to the globe of the eyeball. Distance B represents the diameter of the optic nerve sheath. The illustration was created with BioRender.com.

**Figure 3:**
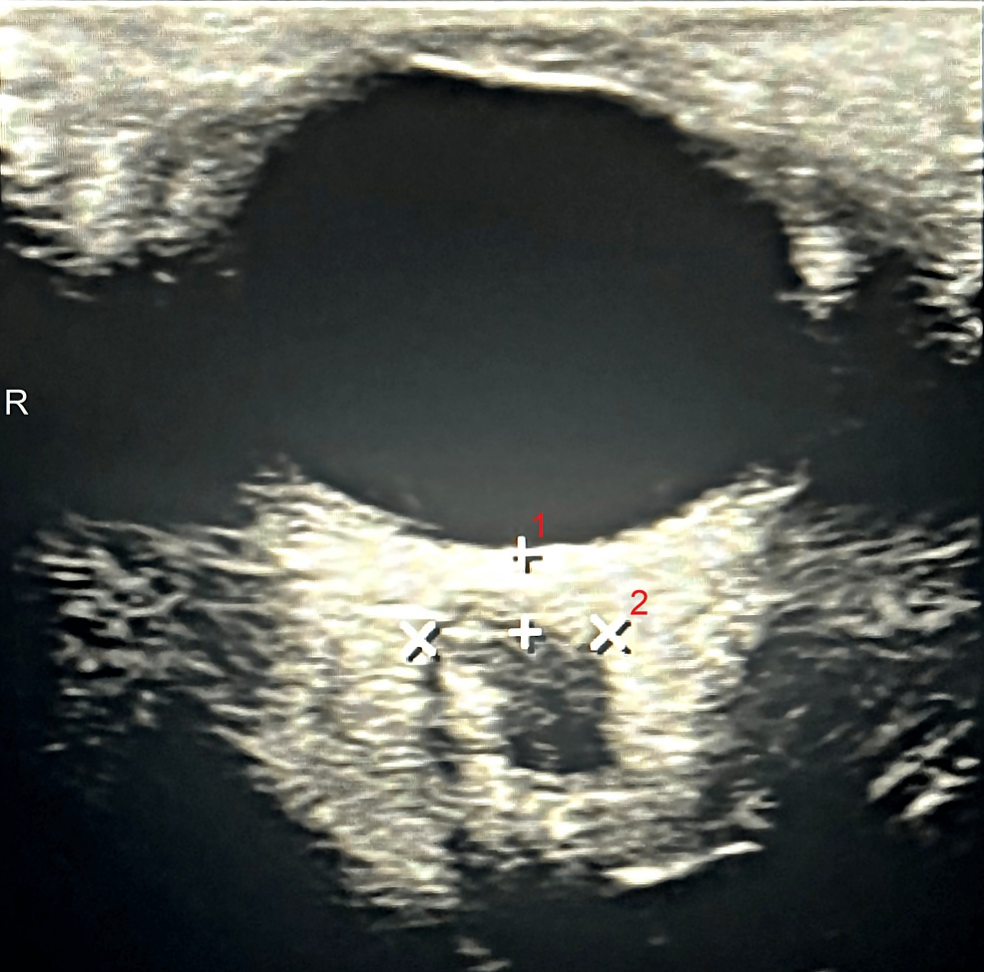
Optic Nerve Sheath Ultrasound The diameter of the optic nerve sheath measured 3 mm posterior to the globe of the eyeball (distance 1). The ultrasound image is obtained via a transverse view through the left eye. The transverse diameter of the optic nerve sheath was found to be 0.738 cm (distance 2).

**Figure 4:**
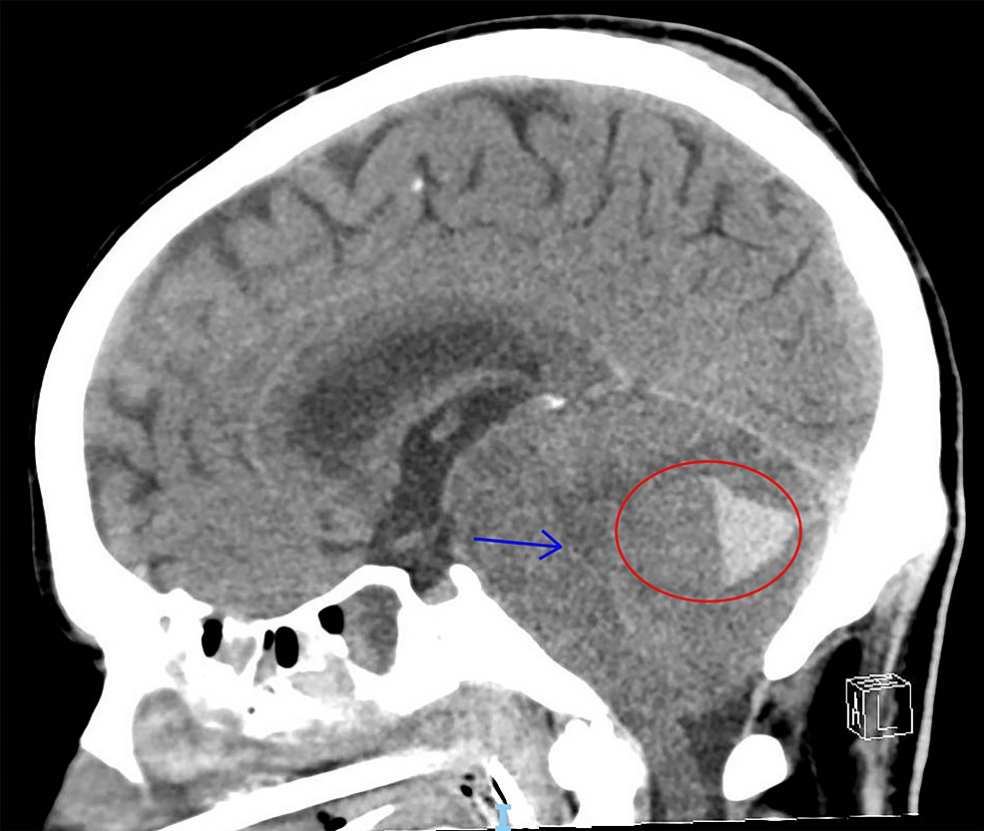
Final CT Head Final CT imaging after the patient was stabilized enough for transportation. Shown is a mid-sagittal slice. Imaging demonstrates enlarging left cerebellar parenchymal hemorrhage (red circle, measured at 5.7 cm x 1.8 cm) with effacement of the basal cisterns and fourth ventricle and brainstem compression (blue arrow).

The following day, he had minimally reactive pupils. Ultrasonography was utilized to assess ONS diameter. The optic nerve sheath was measured with a linear high-frequency (6-13 MHz) ultrasound probe. Tegaderm was applied over closed eyes with adequate ultrasound gel layered above the dressings. The ultrasound probe was then fanned through transverse planes of the eyes, minimizing pressure to the eyes, until the mid-axial planes were identified. With the optic nerves visualized in the mid-axial planes, 3 mm was measured posterior to each globe, followed by measuring the distance across the hypoechoic nerve sheaths (Figure 2). The process was repeated several times for each eye. Over several measurements, the diameter remained above 0.7 cm bilaterally, with a final measurement of 0.738 cm taken for the left optic nerve (Figure 3). In contrast, the upper limit of normal for an adult is between 0.5 - 0.55 cm [6-7]. No immediate action was taken as the patient was not an operative candidate and not a candidate for pausing anticoagulation. Later, in the early morning hours, the patient’s pupils became fixed and dilated, accompanied by hemodynamic instability requiring vasoactive support with norepinephrine up to 12 mcg/min and vasopressin up to 0.04 units/min. After the period of instability had passed, a repeat CT Head was performed which confirmed our suspicions of worsening cerebellar bleeding, measuring 5.7 cm x 1.8 cm. Additionally, an occluded fourth ventricle, obstructive hydrocephalus, and significant brainstem compression were demonstrated (Figure 4). These findings were presented to all the involved services, including neurosurgery, cardiac surgery, critical care/intensivist, and advanced heart failures, as well as to the family. The decision was made to withdraw care and the patient passed away peacefully, with family at the bedside shortly thereafter.

## Discussion

As the optic nerve is continuous with the central nervous system, its meningeal sheath is an extension of the dura mater and will dilate as increasing ICP causes an influx of cerebrospinal fluid. In a meta-analysis performed by Robba et al., the accuracy of an ONS diameter of ≥ 0.6 cm for identifying ICPs > 20 mmHg ranged from 0.811-0.954, with an area under the receiver-operating characteristic curve of 0.938 [7]. Measurements of the optic nerve sheath show linear changes until a plateau of around 0.75 cm, though there is not a direct relationship between ONS diameter and ICP beyond the threshold value [8]. However, additional prospective studies and meta-analyses have corroborated the reliability of ONS ultrasound for detecting elevated ICP, where transverse sheath measurements above 0.55 cm are 90-96% sensitive and 85-92% specific for diagnosing intracranial pressures greater than 20 mmHg [8-9]. Furthermore, Toscano et al. demonstrated that ONS ultrasound has shown utility for the early detection of ICP changes in sedated patients with neurologic critical illnesses [10].

Currently, there is no standardized method of screening ECMO patients for neurologic injury. While the National Institute of Health Stroke Scale has been proposed as a standardized means of detecting focal deficits, this method is not always applicable in the case of insidious onset of injury or the inability to wean sedation [11]. CT remains the predominant method of assessing intracranial pathology while on ECMO; however, while this imaging technique has a high sensitivity for detecting intracranial hemorrhages, the sensitivity is poor in the setting of early ischemic insults. Other proposed methods outside of ONS sonography include cerebral near-infrared spectroscopy, transcranial Doppler, and electroencephalography; however, little data has been collected on these methods and they are not frequently applied in practice [11]. ONS sonography remains uniquely different than CT imaging, which entails a significant investment in labor, equipment, and personnel, as ONS ultrasound can be readily performed within the intensive care unit (ICU). With minimal training, any staff on the clinical care team can be educated to obtain reliable optic nerve measurements [12]. These measurements can then be repeated to trend changes in ICP, providing longitudinal data on a patient’s clinical progression.

While its accuracy in detecting elevated ICP has been validated in neurosurgical ICUs against invasive intracranial pressure monitors such as ventriculostomy and intraparenchymal microtransducers [8-10], sonographic evaluation of the ONS has not been explored in cardiothoracic ICUs. This holds especially true for patients on ECMO where the risk for ICH is high and current diagnostic techniques carry risks for worsened morbidity and mortality. However, the significant limitation of this case report was that an optic nerve ultrasound was not performed prior to the patient's neurologic decline, precluding the ability to trend changes in the diameter as his neuro status deteriorated. Ideally, the ONS diameter would have been obtained on admission, then at the time of initial ICH, and then at a set time thereafter. Further prospective analysis is warranted on whether routine optic nerve ultrasonography for patients sedated on ECMO could improve outcomes by allowing for earlier diagnosis and changes in treatment.

## Conclusions

This case highlights the potential utility of ONS ultrasound in detecting new or worsening changes in intracranial pressures in adult patients on ECMO. With these patients often too unstable for transportation to obtain CT imaging, an alternative, safer tool for guiding care decisions may be optic nerve ultrasonography. While heavily explored in neurological ICUs, to date there have been no published studies analyzing how this methodology could be applied to improve patient care and outcomes in adult cardiothoracic ICUs. Therefore, further prospective data should be collected to evaluate the benefit of including optic nerve sheath ultrasounds as part of the standard assessment for patients on ECMO.
